# Opportunities and challenges of disease biomarkers: a new section in the journal of translational medicine

**DOI:** 10.1186/1479-5876-10-220

**Published:** 2012-11-07

**Authors:** Xiangdong Wang, Peter A Ward

**Affiliations:** 1 Department of Respiratory Medicine, Biomedical Research Center, Zhongshan Hospital Qing-Pu Branch, Fudan University, Shanghai, China; 2 Clinical Bioinformatics, Lund University Hospital, Lund, Sweden; 3 Department of Pathology, University of Michigan Medical School, Ann Arbor, MI, USA

## 

The word “biomarker” describes a traceable and characterized substance that is an indicator of biological morphology, processes and function. Disease biomarkers are used to diagnose various phases of diseases, monitor severities of diseases and responses to therapies, and are predictors of prognosis of patients and likely responses to therapy. One of criteria to evaluate the value of disease biomarkers is the disease-associated specificity, sensitivity, traceability, stability, repeatability and reliability. Increasing numbers of biomarkers are being discovered and identified from preclinical research, but only a few have been found to be useful clinically. The Journal of Translational Medicine opens a new Section of *Disease Biomarkers* to bridge identification and validation of gene or protein-based biomarkers, network biomarkers, dynamic network biomarkers in human diseases, patient phenotypes, and clinical applications. The Section intends to accelerate the discovery and development of human disease-specific biomarkers for the early diagnosis, and monitoring, evaluation of diseases and predictions of responses to therapy. The Section will be an important and critical platform to underscore the significance and value of *Disease Biomarkers* and promote innovation and development of disease-specific biomarkers by integrating multidisciplinary aspects of science. The following are aspects of the planned biomarker studies:

a) Benefitting drug development: Disease biomarkers can play critical roles not only in clinical applications but also in drug discovery and development as related to drug efficacy or toxicity, allowing selection of “the right drugs and the right patients” [1]. For example, more than 90% of new anti-cancer drugs in clinical trials have failed marketing approval, often due to poor efficacies and efficiencies, high toxicities, drug resistance and unexpected safety issues. The lack of disease-specific biomarkers is a major problem, often making it difficult to predict drug efficacy. The Section of Disease Biomarkers will accelerate the use of biomarker identification and validation for determining drug effects, target specificities and binding, dynamic metabolism and pharmacological kinetics, toxicity profiles, and side-effects. In addition, Disease Biomarkers will emphasize the importance of molecular and cellular responses to drugs under pathophysiological conditions, creating a new concept of “the right biomarker, the right drug and the right patient”. This will benefit our understanding of molecular mechanisms of interactions between biomarkers, drugs and diseases. Optimal biomarkers should allow monitoring drug efficacy and safety and individual responses to specific treatments. Patients evaluated and treated at the early stage of disease may enhance business decisions related to drug development, facilitating regulatory approval for new therapies [2]. Quantification and safety of biomarkers are necessary for final decisions related to new drug development and applications.

b) Integration of multidisciplinary sciences: Identification, validation, development and marketing of disease-specific biomarkers are integrated processes in molecular biology, involving new biotechnologies, clinical sciences, regulatory policies, and clinical applications. “Omic” science and technology play important roles in the identification and discovery of biomarkers. The Omic scope includes genomics, proteomics, metabolomics, pharmacogenomics, transcriptomics, and other high-throughput methodologies. Selected biomarker candidates can be validated and evaluated using computational biology, high-throughput image analysis, molecular genetics, specimens from human tissue banks, mathematical medicine and biology, protein expression and profiling, and systems biology. Clinical bioinformatics have been suggested as a new way to combine clinical measurements and signs with human tissue-generated bioinformatics, helping to understand the role of selected biomarker candidates in clinical settings, disease development and progression, and therapeutic strategies, mapping relationships of drug and biomarker candidates with clinical examinations, pathology data, biochemical analysis, and imaging and therapies [3]. An example of targeted biomarker validation has been the study drug localization, is tissues, using imaging data of targeted tumor regions in various lung compartments. Another example is selectivity and precision of proteomic analyses in patients with chronic lung diseases and cancer [4]. Panels of disease-specific protein biomarkers that define the disease stage were selected in chronic obstructive pulmonary disease. By targeted proteomic analysis, a digital evaluation score system was developed for assessing severity of disease, useful, bioinformatics information, and lung function [5,6]. A number of new integrated scientific areas have been created during the identification and development of disease-specific biomarkers. Genomic medicine was proposed to bring biomarkers into the mainstream of clinical practice and improve therapies and quality of patient life, although such strategies are in the very early stages. Systems clinical medicine has been defined as one of new strategic areas for development of disease biomarkers, involving integration of systems biology, clinical phenotypes, high-throughout technologies, bioinformatics and computational science in order to improve diagnosis, prognosis and therapies of diseases [7]. Next generation sequencing for genomic analysis of individual genomes has been used for single nucleotide polymorphism discovery and estimates of allele frequency, gene ontology analysis of the target genes, analysis of the microRNAs expression, sequencing platforms for mRNA biomarker analysis, genome-wide analysis, and the exploration of the proteome, all of which should lead to the identification of useful protein biomarkers.

c) Significance of molecular imaging in validation of biomarkers: It has been suggested that selected biomarker candidates need to be validated in human tissues, (e.g. measuring the expression of targeted mRNA and proteins in pathological tissues of patients) as well as correlating the over-expression of targeted candidates with dysfunction of organs or tissues. Tissue microarray analysis is a powerful tool for validation of biomarker candidates, especially with an algorithm-tissue array co-occurrence matrix analysis for quantifying cellular phenotypes based on textural regularity by local inter-pixel configurations [8]. In addition, matrix-assisted laser desorption/ionization mass spectrometry imaging is used for spatial distribution and relative abundance of biomolecules directly in tissues in order to improve the quality of molecular images and differentiate tissue regions that cannot be morphologically defined. It has been suggested that such imaging may be beneficial for disease diagnosis and prognosis, biomarker discovery and drug therapy, even though there are still many barriers. As an example, clinical phenotypes and severities of chronic obstructive pulmonary disease were measured by the parametric response map, a voxel-wise image analysis technique of whole-lung using computed tomography that results in more accurate diagnosis of individual patients, together with integration of clinical information [9]. Numerous imaging technologies have been applied for biomarker validation and development, including near infrared imaging, neuronuclear imaging, whole-body diffusion magnetic resonance imaging, specific biomarker detection on the cell surface in real time, positron emission tomography, molecular contrast-enhanced ultrasound imaging, and acceptor fluorescence anisotropy that measures variations in hetero- fluorescence resonance energy transfer deriving from protein-protein interactions.

d) Network biomarkers and dynamic network biomarkers: Multiple biomarkers were found to improve the prediction of death from cardiovascular causes during a more than 10 year follow-up of patients, suggesting that simultaneous presence of biomarkers in cardiovascular disease as well as presence of established risk factors substantially improves the risk stratification for death from cardiovascular disease in elderly men [10]. Network biomarkers and dynamic network biomarkers represent new types of biomarkers with protein-protein or gene-gene interactions that can be monitored and evaluated at different stages and time points during development of disease [11]. The amount of high-throughput genomic and proteomic data from patients has been rapidly increasing and is expected to correlate with clinical phenotypes, disease severities, therapeutic responses, and prognosis. Gene regulatory networks or protein interaction networks may describe functions for the panel of relevant network biomarkers. Dynamic network biomarkers can demonstrate changes at various stages in diseases and seem to have disease specificity. Disease biomarkers need to be validated by integration with clinical informatics, which translates clinical descriptive information on signs, symptoms, biochemical analyses, imaging and therapies into digital data [5,6]. Clinical bioinformatics may be helpful in discovering disease-specific, stage-specific, severity-specific and therapy-predictive biomarkers.

e) Challenges in development: As one of major technologies for biomarker identification and discovery, genomics and proteomics as well as sequencing still face a large number of challenges, (e.g., analysis of the individual’s genome in the context of extensive population-based data, phenotypic significance and specificity, duration and severity, of disease, genomic or proteomic variation, combinations of genomic, expression-based, metabolomic, and proteomic data, and the value of disease biomarkers for making clinical decision). Some tissue microarrays have been questioned due to study sizes, reduced throughput, variability, and expenses related to the observations. A recent study combined genome-wide association with forced expiratory volume in 1 second and the ratio of forced expiratory volume in 1 second to forced vital capacity, identified 16 new genomic regions showing association with pulmonary function [12]. Those biomarkers were selected from 48,201 individuals of European ancestry, with follow up of top associations in an additional 46,411 individuals. However, there is further need to better understand the biomarkers associated with molecular mechanisms involving pulmonary function and validate selected targets in order to reduce lung dysfunction.

## Conclusions

The Section of Disease Biomarkers is expected to accelerate the process from identification to validation, research to development, resulting in improved design of clinical trials. There should also be positive impacts on governmental approval, and clinical applications to policy and regulation, and relevance to “personalized” medicine and to public health. The Section will publish articles related to development of advanced biotechnologies for biomarker discovery, the biomarkers associated with the early detection of diseases, and monitoring of disease severity and duration as well as patient responses to therapies, predictions of patient outcomes and life qualities, the clinical trial and evaluation of biomarkers, and regulation and ethics of disease biomarkers.
